# Cross-Sectional Study of Paediatric Preventive Care in Saudi Arabia

**DOI:** 10.1016/j.identj.2026.109730

**Published:** 2026-07-10

**Authors:** Idris Busaily, Anwen Cope, Waraf Al-yaseen, Mohammed Jafer, Nicola Innes

**Affiliations:** a School of Dentistry, College of Biomedical and Life Sciences, Cardiff University, Heath Park, Cardiff, UK; b Department of Preventive Dental Sciences, College of Dentistry, Jazan University, Jazan, Saudi Arabia; c School of Dentistry, University of Leeds, Clarendon Way, UK

**Keywords:** Saudi Arabia, Children, Caries prevention, Guideline adherence, Dentists

## Abstract

**Introduction and aims:**

The high prevalence of dental caries is recognised as a public health challenge in Saudi Arabia. The shift from treatment-based approaches to prevention is one of the objectives to control caries prevalence. Two evidence-based methods to prevent caries are applying fluoride varnish (FV) and fissure sealants (FS). However, data on practices related to their application is limited. Therefore, this study aims to: (1) assess the extent to which General Dental Practitioners (GDPs) in primary healthcare centres in Saudi Arabia provide FV and FS for children aged 14 years and younger; and (2) identify overall preventive measures they deliver in practice.

**Methods:**

A multicentre, prospective, descriptive cross-sectional study was conducted in Jazan province, Saudi Arabia, April to August 2024. Direct clinical observation allowed quantification of GDPs’ application of FV and FS in routine clinical care for children aged 14 years and younger. This was compared against current evidence-based practice guidelines.

**Results:**

Thirty-five GDPs were observed treating 643 children. Although 98% of children met eligibility criteria to receive FV, only 4% received it. Similarly, 74% were eligible for FS, and only 2% received one or more sealants. Caries prevention advice was provided in 80% of consultations, and the most frequently delivered preventive measure was toothbrushing instruction.

**Conclusion:**

Despite high levels of eligibility according to recommendations, there were low delivery rates of FV and FS by GDPs, for children in primary healthcare centres in Saudi Arabia, highlighting the gap between evidence-based guidelines and clinical practice.

**Clinical relevance:**

The finding of this study indicates that the adoption of international guideline recommendations on the use of FV and FS in Saudi Arabia has been insufficient to optimise the delivery of these preventive interventions. This highlights the need for further research to understand the behaviour determinants that influence GDPs in delivering them.

Review retrospectively registered: Open Science Framework https://doi.org/10.17605/OSF.IO/2XS6G.

## Introduction

A recent national report found that approximately 96% of 6-year-old children and 93% of 12-year-old children in Saudi Arabia were affected by dental caries.^1^ These findings are consistent with a systematic review reporting 84% prevalence of dental caries in primary teeth among children aged 5 to 7 years and 72% in permanent teeth of children aged 12 to 15 years.^2^ The high prevalence of dental caries has been recognised as a significant public health challenge for the country. Consequently, prevention has been made a cornerstone of Saudi Arabia’s Vision 2030, which was established in 2016 to reconstruct the government spending and country development.^3^^,^^4^ The shift from curative-based approaches towards a prevention focussed service is now considered one of the main objectives of the Ministry of Health for healthcare providers, with GDPs working in primary healthcare centres part of this transition.^3^^,^^5^

Two of the most common and effective caries preventive measures used by general dental practitioners (GDPs) are fluoride varnish (FV) and fissure sealants (FS).^6^ FV is a topical chemical approach to promote remineralisation, while FS provide a physical barrier between the tooth surface and food/ bacteria, thereby reducing the opportunity for dental caries to establish and progress.^7^^,^^8^ Meta-analyses of 23 clinical trials support the use of FV for both primary and permanent teeth as an effective caries-preventive intervention.^9^ The Scottish Dental Clinical Effectiveness Programme (SDCEP) and European Academy of Paediatric Dentistry (EAPD) recommend at least two FV applications per year for all children of high caries risk.^10^^,^^11^ The most commonly used formulation of FV is 5% sodium fluoride.^12^ FS is an effective, evidence-based intervention for the prevention of caries in both primary and permanent molars in children.^13^ Multiple guidelines strongly recommend its application on newly erupted permanent molars.^8^^,^^10^ It is generally considered that the combined use of both (FV/FS) is likely to have a synergistic effect.^14^

In Saudi Arabia the current regulations-governing-dental-services, issued by the Ministry of Health in 2004, state that GDPs in primary healthcare centres are responsible for providing FV and FS to children free of charge.^3^^,^^15^ Despite these guidelines, there is limited data on the actual practices around FV and FS use in Saudi Arabia. No studies have directly reported GDPs’ use of FV. Parent-reported data indicate that only around 1.7% of children received FV applications from their GDP.^16^ The evidence on FS use is conflicting. GDP-reported application rates range from 28% to 76%, while epidemiological studies indicate that only 1% to 6% of children have sealants.^17^, ^18^, ^19^, ^20^, ^21^ In the wider context of Gulf Cooperation Council countries, two studies have reported GDPs’ use of FV. In Bahrain, 46.3% of GDPs applied FV occasionally and 4.5% rarely.^22^ Whereas in Kuwait, only 3% reported using FV. No clear data were available on the FS application.^23^

Therefore, this study aims to assess the extent to which GDPs in primary healthcare centres in Saudi Arabia provide FV and FS to eligible children 14 years old and younger, and to identify overall preventive measures they currently apply in practice.

## Material and methods

### Study design

This multicentre, prospective, descriptive cross-sectional study was conducted in Jazan, Saudi Arabia, between April and August 2024. The Jazan region was selected for both feasibility and potential generalisability as GDPs in primary healthcare centres across Saudi Arabia work under the same system and provide similar service. Trained observers observed GDPs while they provided routine dental care in their clinics.

### Ethical considerations

The study complied with the ethical principles and ethical approval was obtained from the Ministry of Health in Saudi Arabia Approval No. 2418 (31/01/2024) [Appendix A] and from The Jazan University for the protocol Reference No. REC-45/09/1011 (11 March 2024) [Appendix B]. Written informed consent was obtained from all participating GDPs, as the focus of the study was their behaviour in applying evidence-based preventive care. Children were not enrolled as research participants. No identifiable information was collected from them. Only age and gender were recorded. Parents or legal guardians were informed that an observer would be present and they had the right to decline. No parents or legal guardians refused.

### Participants and recruitment

The target population were GDPs working for the Ministry of Health, in primary health care centres. GDPs were eligible to participate in the study if, at the time of recruitment, they were licensed to practice by the Saudi Dental Licensing Board, employed and practising in primary healthcare centres within Saudi Arabia, providing full or part time clinical care, and available for the full duration of the study. GDPs who did not treat children 14 years old and younger were excluded from the study.

GDPs were identified using a database of registered Ministry of Health General Dental Practitioners working in the Jazan region. According to the statistical yearbook from 2022, there were approximately 318 GDPs, with around half of them working in primary healthcare centres.^24^ Details of practicing GDPs, including the names of the centres where they worked were requested from the Jazan Health Cluster. Each primary healthcare centre was assigned a unique number using a simple sequence.

A simple random sampling approach using a random number generator selected 30 practices from the list.^25^ An email was sent from the Jazan Health Cluster to all selected primary healthcare centres. Following this, All GDPs within selected practices were invited to participate in the study and sent the participant information sheet and consent form through their preferred contact method, either WhatsApp or phone number. Informed written consent was obtained prior to observation.

### Data collection

The case report form (CRF) was designed according to the objectives of this study, the scientific literature, current guidelines, research and clinical experience of the research team [Appendix C].^8^^,^^11^ The CRF comprised 26 questions divided into five sections. The first section included general information such as observer’s number and date of observation. The second section captured demographic information about the child patient, limited to the child’s age and gender. The third section included questions assessing adherence to evidence-based practices, such as taking a history and the processes of asking about, planning, and applying FV and FS. Eligibility criteria for FV are that the child is older than 1 year, while for FS, the first permanent molars are newly erupted, have not been previously received FS and are noncavitated. The fourth section addressed whether caries prevention advice was offered, and the nature of the advice provided. The fifth section included any other observation notes.

Data were collected through observation and documentation by five observers. Each GDP was observed over a few days until the required number of observations^18^, ^19^, ^20^, ^21^, ^22^, ^23^, ^24^, ^25^ was completed. Each observer was assigned to a specific GDP within a particular practice. As the number of child patients varied between practice, some observers completed their observations more quickly and were subsequently reassigned to other practice until observations across all practices were completed. The principal investigator (a Paediatric Dental Specialist) conducted a training and calibration process with the observers; a didactic lecture on the use of FV and FS, completed a CRF for GDPs treating ten cases, with the trainer (considered as gold standard) completing a CRF for comparison across nine key domains. To assess the reliability of the observers, two tests were performed. The Intraclass Correlation Coefficient (ICC) assessed the absolute agreement and consistency between the raters for the continuous data points [Appendix D] with almost absolute agreement (ICC ≈ 1) between the raters and the trainer. Cohen’s Kappa measured agreement between each observer and the trainer for categorical data.^26^ The results ranged between 0.873 and 0.955, indicating excellent agreement [Appendix E].

#### Sample size

The key outcomes on which the sample size calculation were based were the proportions of participating GDPs using professionally applied FV and FS respectively from previous research. This indicated that the application of FV and FS were both low at around 10%.^17^^,^^27^^,^^28^ Based on this expected prevalence and using a 95% confidence interval, a ±10% margin of error and a population of 185 Ministry of Health general dental practitioners in Jazan, Saudi Arabia, it was calculated that observing a minimum of 30 practitioners would provide sufficient statistical power to assess FV and FS application.^29^^,^^30^ This has been calculated using the finite population correction formula^31^:

$$n\;=\frac{N\;\times \;X}{\left(X\;+\;N\;-\;1\right)}$$

$$X\;=\frac{Z_{\alpha /2}^{2}\;\times \;p\left(1-p\right)}{MOE²}$$

*n*: represents the required sample size; *N*: the population size; Z_α/2_: the standard normal deviate corresponding to the selected confidence level; p: the estimated population proportion; MOE: the margin of error.

### Analysis

The analysis was conducted using RStudio.^32^ The demographic characteristics of the GDPs and paediatric patients were summarised by descriptive statistics.

A frequency table was created for number of GDPs who inquired about or recorded prior FV/FS application, or who planned to apply FV/FS and crosstabulations to explore the proportion of eligible children who received FV and FS. Descriptive statistics were used to describe the distribution of GDPs offering caries prevention advice.

A generalised linear mixed model (GLMM) with a logistic link function was conducted to examine the likelihood of FV or FS being applied. The model accounted for the clustered nature of the data; multiple children treated by the same GDP. It also examined the effects of practitioner- and patient-level factors (including the GDP’s years of experience and gender, and the patient’s gender and age) on the application of FV and FS for eligible children (binary outcome).^6^^,^^8^^,^^33^, ^34^, ^35^ Likelihood ratio tests were performed to select the best-fitting model which included Akaike Information Criterion (AIC) and Bayesian Information Criterion (BIC).^36^ Statistical significance was set at *P* < .05, and analyses were conducted using complete case analysis. The complete case deletion method was used, whereby cases with missing data were reported but not included in the analysis.

## Results

Each GDP (*n* = 35) was observed between 18 and 25 times, except for five who were observed fewer than 10 times and one GDP, 30 times. The characteristics of the participant GDPs are presented in Table 1. In total, 643 observations were made of children receiving treatment. The mean age of the children was 8 years and slightly more than half (50.5%) were male.

**Table 1:** GDP participants’ characteristics.

Participants	Characteristic	Frequency *n* (%)
GDPs (*n* = 35)	**Gender**	
	Male	23 (65.7)
	GDP**s’ Experience**	***Years***
	Range	5 to 14
	Mean (Standard Deviation (SD))	8 (2.2)

GDP’s recording and planning for FV and FS for child patients are reported in Table 2. In 6.5% of consultations GDPs planned to apply FV and in 3.1% of consultations they planned to apply FS. Data of the plan application were not collected for the noneligible cases (ie, missing by design).

**Table 2:** Summary of GDPs’ records of previous FS and FV applications and planned future applications.

		Child visits *N* = 643 (%)			
	GDPs (*N* = 35)	Yes (%)	No (%)	Not sure (%)	Total (%)
FV	GDP inquired about previous FV application	18 (2.8)	625 (97.2)	0	643 (100)
	GDP planned to apply FV†	42 (6.5)	589 (73.9)		631 (98.1)†
FS	GDP inquired about previous FS application	41 (6.4)	601 (93.5)	1 (0.2)	643 (100)
	GDP planned to apply FS*	20 (3.1)	459 (71.3)		479 (74.5)*

† Data on the application plan were not collected for the noneligible cases for FV (*n* = 12).

⁎ Data on the application plan were not collected for the noneligible FS cases (*n* = 164).

The number of children eligible for preventive measures, and who received them during the consultation, are presented in Table 3. In total 98.1% of children (*n* = 631) were eligible to receive FV, and 4% (*n* = 26) received it. Similarly, 73.9% were eligible for FS (*n* = 475), and 1.9% received one or more (*n* = 12). The total noneligible children for FV were 1.9% (*n* = 12). Of these, 0.6% (*n* = 4) did not receive FV, while data for the remaining cases 1.2% (*n* = 8) were missing by design. Likewise, 25.7% (*n* = 165) of children were not eligible for FS. Among these, 0.2% (*n* = 1) received FS, 16.2% (*n* = 104) did not receive FS, and 9.3% (*n* = 60) had provision data missing by design.

**Table 3:** Frequency of children eligible for FS/FV and corresponding application status (*N* = 643).

			FS/FV applied			
			Yes (%)		No (%)	Total (%)
FV	Eligibility†	Yes		26 (4.0)	605 (94.1)	631 (98.1)
		No		0 (0.0)	4 (0.6)†	4 (0.6)
	Total			26 (4.0)	609 (94.7)	635 (98.8)
FS	Eligibility*	Yes		12 (1.9)	463 (72.0)	475 (73.9)
		No		1 (0.2)	104 (16.2)*	105 (16.3)
		Not sure		0 (0.0)	3 (0.5)	3 (0.5)
	Total			13 (2.0)	570 (88.6)	583 (90.6)

† Eight noneligible cases (1.2%) are missing data regarding the application of FV.

⁎ Sixty noneligible cases (9.3%) are missing data regarding the application of FS.

**Fig. 1:**
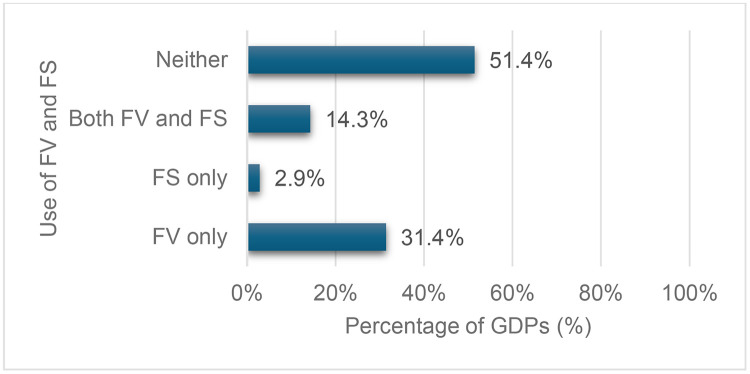
Percentage of GDPs using FV and FS (*n* = 35).

Of the 35 participating GDPs, six applied FS. Within this subset of practitioners, the use of FS was low, with the most active practitioners applying them to 22% of eligible patients. Similarly, 16 GDPs applied FV with the most active practitioner applying them to 20% of eligible children. Eleven of the GDPs applied FV only, one applied FS only, five applied both FV and FS, and 18 applied neither (Figure 1).

In the GLMM model (Table 4) child’s age was the only characteristic significantly associated with FV application (Odds Ratios (OR) 0.72 (95% Confidence Interval (CI) 0.59 to 0.87), *P* < .001). The mean age among children receiving FV was 6.8 years (SD = 2.37), compared to 8.3 years (SD = 2.35) for children not receiving FV. Median ages were 6.5 and 8 years, respectively. Associations between child’s gender, GDP’s experience and GDP’s gender and FV were not statistically significant (*P* > .05). None of the child patient or practitioner characteristics were associated with FS placement (*P* > .05). Although the child age–only model had slightly better AIC and BIC values, the full GLMM was retained because likelihood ratio tests indicated no significant improvement in model fit, and the key findings remained unchanged.

**Table 4:** Generalised linear mixed model (GLMM) analysis of predictors of GDPs’ use of FV/FS.

	Fluoride Varnish†			Fissure Sealant*		
Predictors	OR	CI	*P*	OR	CI	*P*
**Child’s age (Years)**	0.71	[0.59, 0.87]	<.001	0.74	[0.47, 1.16]	.19
**Child’s gender [**M**ale]**	0.96	[0.42, 2.18]	.92	0.35	[0.09, 1.39]	.14
**GDP’s experience (Years)**	1.05	[0.82, 1.34]	.69	1.51	[0.79, 2.89]	.22
**GDP’s gender [**M**ale]**	1.03	[0.32, 3.33]	.96	3.85	[0.15, 99.81]	.42

Note: Reference categories were female GDP and female child patient.

† AIC 213.1 BIC 239.7.

⁎ AIC 101.8 BIC 126.8.

**Fig. 2:**
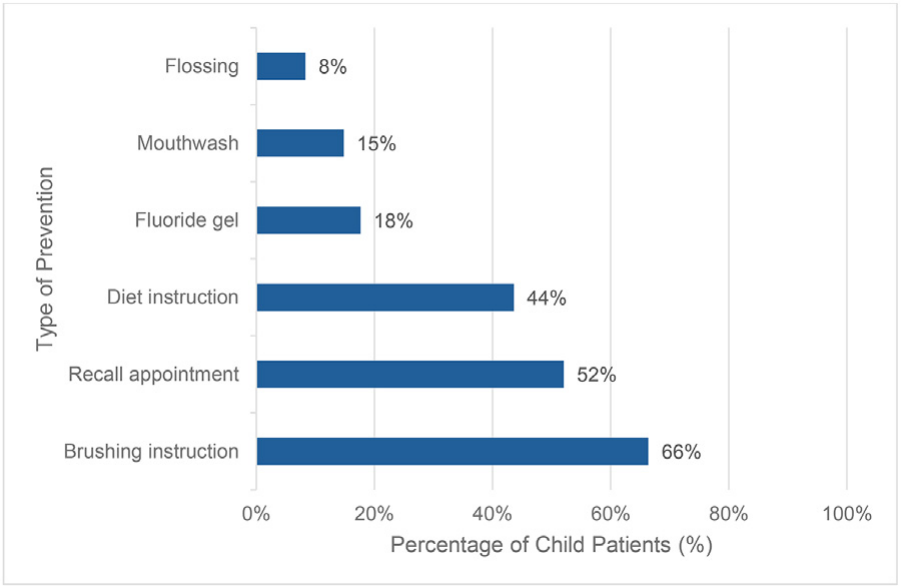
The frequency of different preventive methods delivered/planned by the GDP during child visit (*N* = 643).

GDPs offered advice on caries prevention in the majority of consultations (*n* = 514; 79.9%). The most common forms of advice included toothbrushing instructions, recall appointment intervals, and dietary guidance (Figure 2).

## Discussion

This study using direct observation of GDPs’ activities highlights a substantial gap between international guidelines for delivery of FV and FS and actual delivery of these preventive interventions for children in primary healthcare centres in Saudi Arabia. Despite strong evidence supporting the use of both interventions in children, the majority of practitioners (18/35; 51.4%) apply neither FV nor FS. This underutilisation is concerning, given the high prevalence of dental caries among children in Saudi Arabia.^2^

The rate of FV application during the study observation periods was low (4%). The finding is consistent with previous parental reports indicating limited FV application for children in Saudi Arabia.^16^ Also, similar patterns have been reported in other Gulf Cooperation Council countries.^22^^,^^23^ In international setting, around 80% of children’s treatment plans in Wales include FV application.^37^ Although the delivery of FV is generally high in the UK, many children do not receive the intended number of applications per year.^38^ One study found that only 3.5% of children received 10 or more FV applications over a 10-year period at the GDP.^38^ In Scotland, the Childsmile programme reported an improvement in the proportion of children aged five to eight who received applications from 14% in 2010 to 30% in 2024.^39^^,^^40^ These improvements highlight the importance of using a theory-based approach and evidence-based health improvement activities, which can be applied in different context such as in Saudi Arabia.^41^

Similarly, the rate of FS application was similar to those reported in previous studies conducted in Saudi Arabia.^17^^,^^18^ Alwayli et al^17^ found that 1.3% of 17,891 schoolchildren had at least one sealed tooth. In another study, 9% of 1668 third- and eighth-grade students had FS.^18^ In contrast, data from the United States show a continuous improvement in the provision of FS to children.^42^ The proportion of children with sealants increased from 31% during 1999-2004 to 42% during 2011-2016.^42^ These improvements were likely due to increased access to preventive care services through changes of insurance programme policies.^43^^,^^44^ The proportion of children who had public insurance and received preventive dental treatment increased from 23% to 49% between 2000 and 2020.^43^ Additionally, the inclusion of preventive care within primary medical care settings, schools, and community programmes along with the promotion of dental visits for young children at risk of early childhood caries has contributed to this positive trend.^45^^,^^46^ In Denmark, a study involving 3184 15-year-olds reported that two-thirds of the children had dental sealants.^47^

Examining the factors that may influence GDPs’ delivery of FV or FS to children, child’s age was the only significant factor for FV application, with younger children being more likely to receive it than older children. Similar results were found in Scotland, where GDPs reported applying FV more often for younger children (3-5-year-olds).^48^ However, the child’s gender, GDP’s gender, and years of experience did not significantly influence GDP’s application FV or FS, suggesting other factors might influence their behaviour.

Studies in different countries have identified that barriers to guideline adherence occur at multiple levels, such as system, behavioural (at practitioner level) and parental barriers.^49^^,^^50^ At the system level, there is insufficient integration of guidelines into primary dental care, inadequate reimbursement for prevention, limited time, and limited healthcare policy support for prevention.^21^^,^^51^, ^52^, ^53^ A recent study highlighted the potential for integrated digital tools into GDPs’ clinical practice.^54^ Behavioural barriers amongst practitioners include a lack of confidence in the effectiveness of preventive interventions, a lack of knowledge of indication for their use, and a tendency to prioritise treatment over prevention.^20^^,^^23^^,^^51^ A study in Saudi Arabia found that many GDPs did not place FS due to concerns about sealing over caries, despite evidence supporting its safety and efficacy in sealing noncavitated dental caries.^55^

Limited parental awareness, demand and also lack of knowledge and understanding of the importance of their child’s oral health have been cited as barriers to the delivery of preventive dental care in the literature.^51^^,^^56^ Low-income families struggle with dental care because of their inability to cover the costs and the absence of dental coverage.^57^ Multiple studies have cited language barriers as a factor negatively influencing the quality of dental care.^57^^,^^58^

The key strength of this study is its prospective in nature and focus on capturing real-world clinical practice in primary healthcare centres through observation. It provides an overview of current preventive practices and insights into the factors that may influence the delivery of FV and FS in this setting. The low rate of FS application observed in this study (2%) stands in stark contrast to figures reported in previous research (28%-38%) in which GDPs self-reported their use of FS.^20^^,^^21^ However, these studies were conducted 15 to 25 years ago and may not reflect current clinical practice. Also, the discrepancy may reflect differences between self-reported behaviour and actual clinical practice, highlighting the potential influence of social desirability bias or overestimation associated with other methodologies.^59^

Whilst there is a possibility of behavioural modification due to the Hawthorne effect (in this case, GDPs knowing they were being observed), this was minimised by having dental interns, who are routinely present in these clinics, conduct the observations.^60^ GDPs were aware that their behaviour was being observed. The consent form specified that preventive dental procedures included oral hygiene instruction, professionally applied FV, FS, dietary advice, and other procedures. This awareness may have influenced the overall provision of preventive care. Caries prevention was provided in 80% of consultations. Although, the true value of FS and FV application may be even lower than we observed. Observing practitioners for a short duration may not fully capture the scope of their preventive practices. Moreover, the scheduling system in primary health care centres did not differentiate between routine and emergency appointments. According to recent systematic reviews, most patients attended due to pain, whereas preventive motives were less common, which may have influenced the generalisability of our findings.^61^ However, the repeated observations provide valuable insight into the routine preventive care delivered to child patients. The low application rates of FV and FS for eligible children also reassure that even if the GDPs were changing their behaviours to what was expected according to clinical recommendations, this was not to any great extent. Although only GDPs working in the Jazan region were included in this study, all GDPs employed in primary healthcare centres across the country work under the same organisation, the Ministry of Health, and provide similar dental services to the general population, with comparable workload patterns. In addition, the sample size calculation and the random selection from the defined sampling frame were performed to enhance the generalisability of our data. Nevertheless, we acknowledge that GDPs working in other governmental or private sectors were not included, and their practice behaviours may differ.

These findings suggest that the adoption of international guideline recommendations on FS and FV use in Saudi Arabia, has been insufficient to optimise the delivery of these preventive interventions. Further research to understand the barriers and facilitators that influence GDPs in Saudi Arabia to deliver preventive dental care to children is needed. Qualitative studies involving in-depth interviews with GDPs and stakeholders could yield rich data on the determinants of provision of prevention measures to children. Policymakers should prioritise the development of evidence-based recommendations aimed at increasing the provision of preventive measures but follow-up work should also explore how effective these are in influencing behaviour change among GDPs to adhere to clinical recommendations and also the ultimate outcome; reduction in dental caries’ rates in children and across their life course.

## Conclusion

This study provides evidence that there is low adherence to evidence-based preventive strategies (FV and FS) for children in primary healthcare centres by GDPs in Saudi Arabia. This is particularly concerning given the high prevalence of dental caries in the country. The child’s age was the only significant factor related to FV application with younger children, more likely to receive FV. These findings highlight the need for new strategies to reduce the gap between the current preventive practice and evidence-based guidelines based on comprehensive understanding of the behavioural determinants underlying FV and FS application.

## Consent for publication

Not applicable.

## Author contributions

The authors’ contributions to the research are as follows: Design of the research: IB, AC, WA, NI. Acquisition of data: IB, AC, WA, MJ, NI. Analysis and interpretation of data: IB, AC, WA, MJ, NI. Drafting the manuscript, reviewing, final approval of the version to be published, and agreement to be accountable for all aspects of the work: IB, AC, WA, MJ, NI.

## Ethics approval and consent to participate

The study complied with the ethical principles and ethical approval was obtained from the Ministry of Health in Saudi Arabia Approval No. 2418 (31/01/2024) and from The Jazan University for the protocol Reference No. REC-45/09/1011 (11 March 2024). The participating dentists received an information sheet and a consent form. The form included details about the purpose and description of the research, potential risks and benefits, and stated that participation was voluntary. Participants were informed that they could withdraw from the study within 14 days following the observation, and any data collected from those who withdrew would be excluded from the analysis.

## Funding

This research did not receive any specific grant from funding agencies in the public, commercial, or not-for-profit sectors.

## Data availability

The datasets generated and analysed during the current study are not publicly available as they form part of the corresponding author’s ongoing thesis. However, they can be obtained from the corresponding author upon reasonable request.

## Conflict of interest

None disclosed.

## Appendix Supplementary materials

Image, application 1

## References

[1] Saudi Ministry of Health (2024). https://www.moh.gov.sa/en/awarenessplateform/OralHealth/Pages/DentalCaries.aspx.

[2] Adam T.R., Al-Sharif A.I., Tonouhewa A., AlKheraif A.A. (2022). Prevalence of caries among school children in Saudi Arabia: a meta-analysis. Adv Prev Med.

[3] Al Saffer Q., Al-Ghaith T., Alshehri A. (2021). The capacity of primary health care facilities in Saudi Arabia: infrastructure, services, drug availability, and human resources. BMC Health Serv Res.

[4] Ministry of Health, Kingdom of Saudi Arabia. HSTP guide [Internet]. 2024 . Available from: https://www.moh.gov.sa/en/Ministry/vro/Pages/manual.aspx Accessed 12 September 2025.

[5] Ministry of Health KoSA (2024). https://www.moh.gov.sa/en/Ministry/vro/Corporatization/Pages/default.aspx.

[6] Dawett B., Deery C., Banerjee A., Papaioannou D., Marshman Z. (2022). A scoping literature review on minimum intervention dentistry for children with dental caries. Br Dent J.

[7] Munteanu A., Holban A-M, Păuna M-R, Imre M., Farcașiu A-T, Farcașiu C. (2022). Review of professionally applied fluorides for preventing dental caries in children and adolescents. Appl Sci.

[8] Wright J.T., Crall J.J., Fontana M. (2016). Evidence-based clinical practice guideline for the use of pit-and-fissure sealants: a report of the American Dental Association and the American Academy of Pediatric Dentistry. J Am Dent Assoc.

[9] Marinho V.C., Worthington H.V., Walsh T., Clarkson J.E. (2013). Fluoride varnishes for preventing dental caries in children and adolescents. Cochrane Database Syst Rev.

[10] Scottish Dental Clinical Effectiveness Programme (SDCEP). Prevention and management of dental caries in children [Internet]. 3rd ed. Dundee: SDCEP; 2025. Available from: https://www.childcaries.sdcep.org.uk/. Accessed 12 September 2025.

[11] Toumba K., Twetman S., Splieth C., Parnell C., Van Loveren C., Lygidakis N. (2019). Guidelines on the use of fluoride for caries prevention in children: an updated EAPD policy document. Eur Arch Paediatr Dent.

[12] American Academy of Pediatric Dentistry. Fluoride therapy [Internet]. Chicago (IL): American Academy of Pediatric Dentistry; 2023. Available from: https://www.aapd.org/globalassets/media/policies_guidelines/bp_fluoridetherapy25.pdf. Accessed 12 September 2025.

[13] Ahovuo-Saloranta A., Forss H., Walsh T., Nordblad A., Mäkelä M., Worthington H.V. (2017). Pit and fissure sealants for preventing dental decay in permanent teeth. Cochrane Database Syst Rev.

[14] Kashbour W., Gupta P., Worthington H.V., Boyers D. (2020). Pit and fissure sealants versus fluoride varnishes for preventing dental decay in the permanent teeth of children and adolescents. Cochrane Database Syst Rev.

[15] Ministry of Health (2004).

[16] Almehmadi A.H., Bannan A., Ahmad A., Alqadi R., Alhindi A. (2022). Parental knowledge and awareness of fluoride varnish application on their children: a cross-sectional study. Int J Gen Med.

[17] Alwayli H.M., Alshiha S.A., Alfraih Y.K., Hattan M.A., Alamri A.A., Aldossary M.S. (2017). A survey of fissure sealants and dental caries prevalence in the first permanent molars among primary school girls in Riyadh, Saudi Arabia. Eur J Dent.

[18] Al Agili D.E., Niazy H.A., Pass M.A. (2012). Prevalence and socioeconomic determinants of dental sealant use among schoolchildren in Saudi Arabia. East Mediterr Health J.

[19] Asali A.T., Pullishery F., Taneja V. (2023). Dentists’ utilization of caries risk assessment and individualized caries prevention methods in pediatric patients in Saudi Arabia. J Pharm Bioallied Sci.

[20] Farsi N.M. (1999). The effect of education upon dentists’ knowledge and attitude toward fissure sealants. Education.

[21] Togoo R.A., Al-Rafee M.A., Kandyala R., Luqam M., Al-Bulowey M.A. (2012). Dentists’ opinion and knowledge about preventive dental care in Saudi Arabia: a nationwide cross-sectional study. J Contemp Dent Pract.

[22] Alrowaili E.F. (2021). Self-reported knowledge about dental caries at young age and variations between dental practitioners in the Ministry of Health in Bahrain. BDJ Open.

[23] Akbar A.A., Al-Sumait N., Al-Yahya H., Sabti M.Y., Qudeimat M.A. (2018). Knowledge, attitude, and barriers to fluoride application as a preventive measure among oral health care providers. Int J Dent.

[24] Ministry of Health SA (2022).

[25] Randomness and Integrity Services Ltd. RANDOM.ORG: True random number service [Internet]. Dublin: Randomness and Integrity Services Ltd. Available from: https://www.random.org/. Accessed 3 February 2024.

[26] Vieira S.M., Kaymak U., Sousa J.M. (2010). In: 2010 IEEE International Conference on Fuzzy Systems (FUZZ-IEEE).

[27] AlHumaid J., Salloot Z., Al-Ansari A., El Tantawi M., AlYousef Y., Al-Harbi F. (2018). Contribution of preventive methods in controlling caries among Saudi primary schoolchildren: a population-based cross-sectional study. Acta Odontol Scand.

[28] Albaeejan F., Bakhashwain D., Alsubaie A. (2021). Sealant utilization and its influence on caries reduction in first permanent molars in Saudi Female School Children. Open Dent J.

[29] Ministry of Health, Kingdom of Saudi Arabia. Statistical yearbook 2022 [Internet]. Riyadh: Ministry of Health; 2022. Available from: https://www.moh.gov.sa/en/Ministry/Statistics/book/Documents/Statistical-Yearbook-2022.pdf. Accessed 28 November 2023.

[30] Lwanga S.K., Lemeshow S. (1991).

[31] Daniel WW. (2009). Biostatistics: a foundation for analysis in the health sciences.

[32] R Core Team (2025).

[33] McKay J.C., Quiñonez C.R. (2012). The feminization of dentistry: implications for the profession. J Can Dent Assoc.

[34] Sangalli L., Souza L., Letra A., Shaddox L., Ioannidou E. (2023). Sex as a biological variable in oral diseases: evidence and future prospects. J Dent Res.

[35] Suga U.S.G., Terada R.S.S., Ubaldini A.L.M. (2014). Factors that drive dentists towards or away from dental caries preventive measures: systematic review and meta summary. PLoS One.

[36] Vrieze S.I. (2012). Model selection and psychological theory: a discussion of the differences between the Akaike information criterion (AIC) and the Bayesian information criterion (BIC). Psychol Methods.

[37] Welsh Government. NHS dental services: April 2023 to March 2024 [Internet]. Cardiff: Welsh Government; 2024. Available from: https://www.gov.wales/sites/default/files/pdf-versions/2024/10/4/1730367040/nhs-dental-services-april-2023-march-2024.pdf. Accessed 31 July 2025.

[38] Brown N., Foley C., Flanagan C., Fujita T., Harford S. (2024). Fluoride varnish applications provided in general dental practice for children of primary school age in three areas of the UK. Br Dent J.

[39] Childsmile Central Evaluation and Research Team, University of Glasgow. Childsmile national headline data [Internet]. Glasgow: University of Glasgow; 2015. Available from: https://www.childsmile.nhs.scot/wp-content/uploads/5225-Childsmile-National-Headline-Data-Nove-2015.pdf. Accessed 29 October 2025.

[40] Childsmile Central Evaluation and Research Team, University of Glasgow. Childsmile national headline data [Internet]. Glasgow: University of Glasgow; 2024. Available from:https://www.childsmile.nhs.scot/wp-content/uploads/Childsmile_National_Headline_Data_Report-December-2024.pdf. Accessed 29 October 2025.

[41] Macpherson L.M., Rodgers J., Conway D.I. (2019). Childsmile after 10 years part 2: programme development, implementation and evaluation. Dent Update.

[42] Centers for Disease Control and Prevention. Oral health surveillance report: trends in dental caries and sealants, tooth retention, and edentulism, United States, 1999–2004 to 2011–2016 [Internet]. Atlanta (GA): US Department of Health and Human Services; 2019. Available from: https://www.cdc.gov/oral-health/media/pdfs/Oral-Health-Surveillance-Report-2019-h.pdf. Accessed 6 July 2025.

[43] Centers for Medicare & Medicaid Services (2023).

[44] Steinmetz E, Bruen BK, Ku L. Children's use of dental care in Medicaid: federal fiscal years 2000-2012 [Internet]. Washington (DC): George Washington University; 2014. Available from: https://www.medicaid.gov/medicaid/benefits/downloads/dental-trends-2000-to-2012.pdf. Accessed 6 July 2025.

[45] Gooch B.F., Griffin S.O., Gray S.K. (2009). Preventing dental caries through school-based sealant programs: updated recommendations and reviews of evidence. J Am Dent Assoc.

[46] American Academy of Pediatrics. Periodicity schedule (Bright Futures/AAP recommendations for preventive pediatric health care) [Internet]. Itasca (IL): American Academy of Pediatrics; 2025. Available from: https://publications.aap.org/pediatriccare/pages/periodicity-schedule. Accessed 8 October 2025.

[47] Ekstrand KR, Martignon S, Christiansen ME (2007). Frequency and distribution patterns of sealants among 15-year-olds in Denmark in 2003. Community Dent Health.

[48] Gnich W., Bonetti D., Sherriff A., Sharma S., Conway D.I., Macpherson L.M. (2015). Use of the theoretical domains framework to further understanding of what influences application of fluoride varnish to children’s teeth: a national survey of general dental practitioners in Scotland. Community Dent Oral Epidemiol.

[49] Templeton A.R., Young L., Bish A. (2015). Patient-, organization-, and system-level barriers and facilitators to preventive oral health care: a convergent mixed-methods study in primary dental care. Implement Sci.

[50] Goff S.L., Gilson C.F., DeCou E. (2024). Barriers and facilitators to optimal fluoride varnish application. Acad Pediatr.

[51] Leggett H., Vinall-Collier K., Csikar J., Owen J., Edwebi S., Douglas G. (2024). A scoping review of dental practitioners’ perspectives on perceived barriers and facilitators to preventive oral health care in general dental practice. BMC Oral Health.

[52] Lienhart G., Elsa M., Farge P., Schott A-M, Thivichon-Prince B., Chanelière M. (2023). Factors perceived by health professionals to be barriers or facilitators to caries prevention in children: a systematic review. BMC Oral Health.

[53] Alkhtib A.O., Ali K., Sajnani A.K., Anweigi L. (2023). Barriers and enablers for oral health promotion programs amongst primary healthcare stakeholders in Qatar – a qualitative investigation. BMC Oral Health.

[54] Teoh L., Biezen R., Taylor M. (2026). Acceptability and usability of a digital medicines tool for dental practitioners in four Southeast Asian Countries. Int Dent J.

[55] Abdulrahman S. (2021). Knowledge and practice of fissure sealants among dentists, dental students, and interns in Saudi Arabia. EC Dent Sci.

[56] Sabbagh H.J., Alghamdi D.S., Almutairi W.M., Alshahrani S.A., Alghamdi A.S. (2019). Knowledge and practices for early childhood caries prevention among parents of the Children Visiting King Abdulaziz University pediatric dental clinics, Kingdom of Saudi Arabia. J Contemp Dent.

[57] Horowitz A.M., Kleinman D.V., Child W., Radice S.D., Maybury C. (2017). Perceptions of dental hygienists and dentists about preventing early childhood caries: a qualitative study. Am Dent Hyg Assoc.

[58] Holland N. (2021). C.E. credit. A historical overview of language access in dentistry: the impact of language access protections on oral health care. J Calif Dent Assoc.

[59] Gordon R.A. (1987). Social desirability bias: a demonstration and technique for its reduction. Teach Psychol.

[60] Choi W.J., Jung J.J., Grantcharov T.P. (2019). Impact of Hawthorne effect on healthcare professionals: a systematic review. Univ Tor Med J.

[61] Aljohani K., Alqarni A., Alshammari A.F. (2025). Reasons for first dental visit in Saudi Arabia: a systematic review. Patient Prefer Adherence.

