# Supplementary material for: Cross-Sectional Study of Paediatric Preventive Care in Saudi Arabia
Source: Int Dent J. 2026 Jul 10;76(5):109730. doi: 10.1016/j.identj.2026.109730 (PMC13380528; doi:10.1016/j.identj.2026.109730)
Supplement: Supplementary file 1 [file mmc1.docx]

# Appendices

## Appendix A: Ministry of Health ethical approval letter


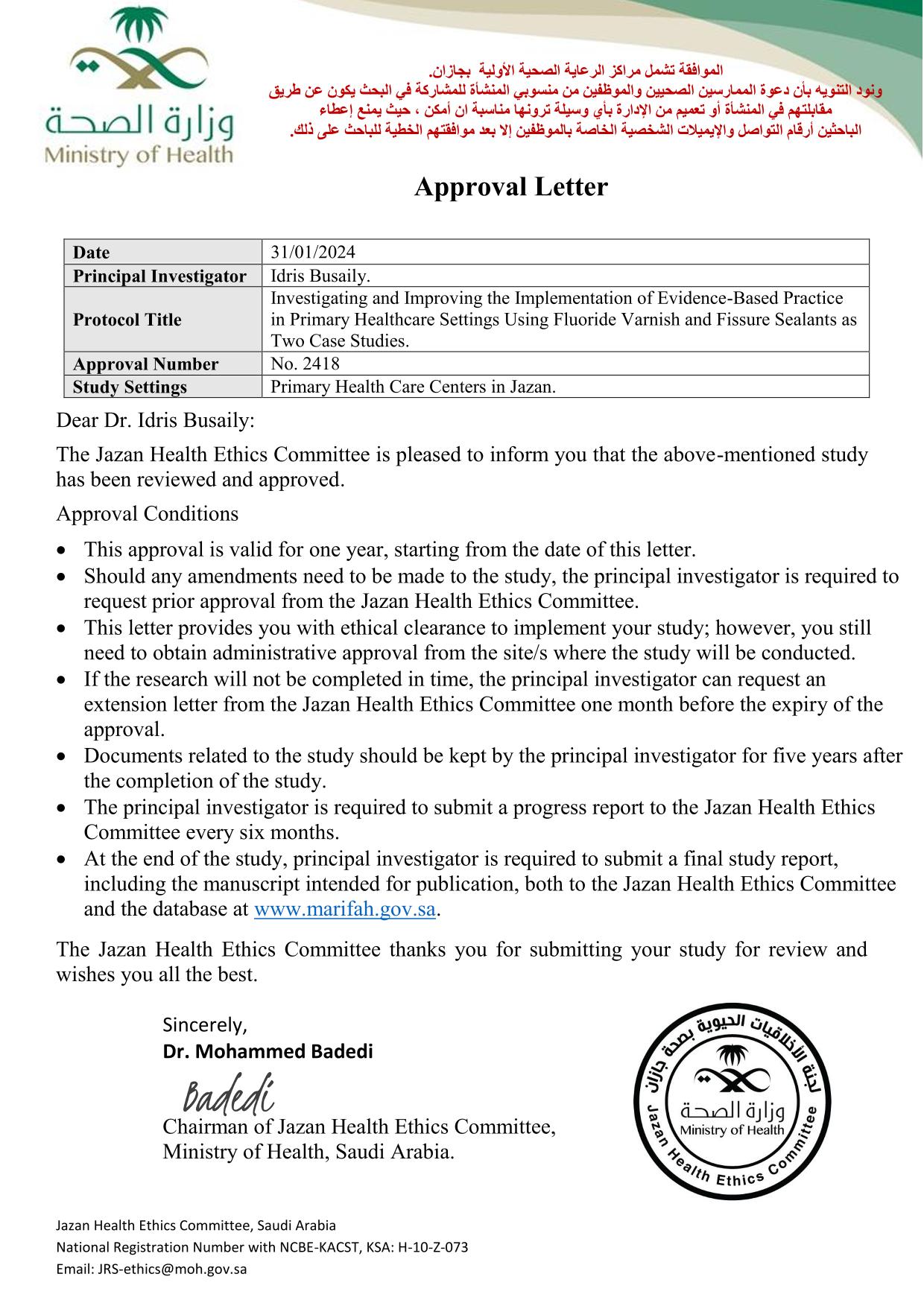


## Appendix B: Jazan University ethical approval letter:


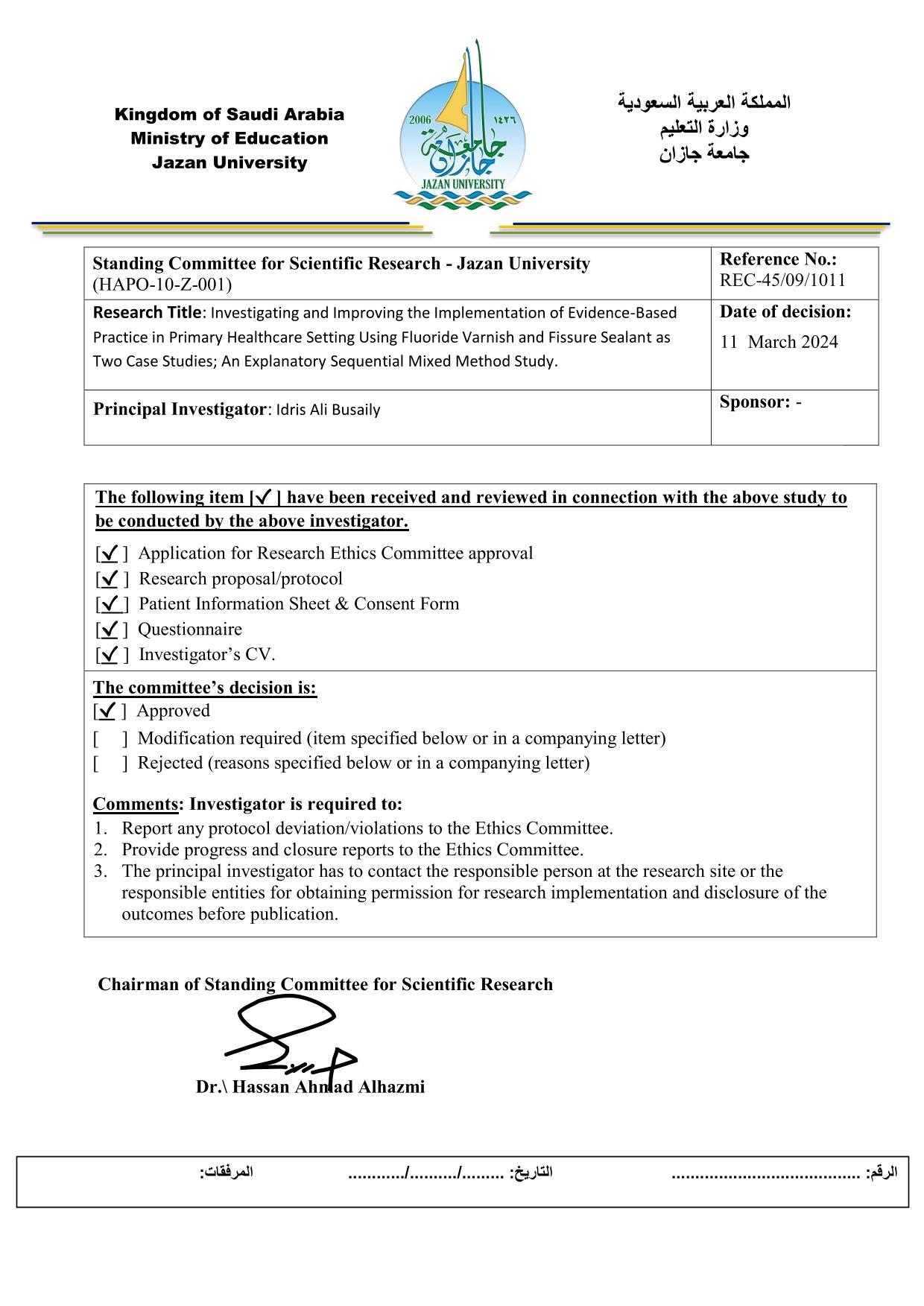


## Appendix C: Case Report Form (CRF)

Dentist Adherence to Evidence-Based Practices

**General Information**

1. Observer’s Name: *
2. Date of Observation *
3. Location/Practice Name *
4. Dentist’s ID *
5. Dentist's gender * Male Female Prefer not to say
6. Graduated from *
7. Dentist’ graduation date *

**Pre-Observation Checklist**

1. Ethical considerations reviewed (e.g., privacy measures) * Yes No
2. Patient’s Age *
3. Patient’s Gender * Girl Boy Prefer not to say

**Assess Adherence to Evidence-Based Practices**

1. Did the dentist take the child history regarding previous hospitalisation because of asthma or allergy to plaster before application? *

Yes/ No

1. Did the dentist inquire or have records of the previous fissure sealant application? *

Yes/ No/ I can't tell

1. Is the patient eligible for fissure sealants according to current evidence-based practice for one tooth at least (newly erupting first permanent molars/ No previous application/ non cavitated dental caries permanent molars)? *

Yes/ No/ Not sure

1. Did the dentist plan or give an appointment to apply fissure sealant to eligible teeth? *

Yes/ No

1. Did the dentist apply fissure sealant to eligible teeth? *

Yes/ No/ Some teeth

1. If yes, which teeth did the dentist apply fissure sealant to

(check all applied)?

Lower permanent molars/ Upper permanent molars/ Lower primary molars/ Upper primary molars

Lower premolars/ Upper premolars

1. What type of material did the dentist use?

Resin-based sealant/ Glass ionomer sealant/ Other

1. Did the dentist inquire or have records of the last fluoride varnish application? *

Yes/ No

1. Is the patient eligible for fluoride varnish according to current evidence-based practice (Child older than 1 year)? *

Yes/ No

1. If yes, did the dentist schedule or recommend a 2nd application within the year as per evidence-based practice? *

Yes/ No

1. Did the dentist apply fluoride varnish during the visit? *

Yes/ No

1. If yes, was the application as per evidence-based practice (appropriate material NaF 22600ppm, clean the clear debris on teeth surface)?

Yes/ No/ Partially, elaborate below in (Other)/ Other

**Cries prevention advice**

1. Did the dentist offer advice on caries prevention? *

Yes/ No

1. If yes, which of the following preventive measure does the dentist discussed

Fissure sealant/ Fluoride varnish/ Brushing instruction/ Diet instruction/ Mouthwash/

Recall appointment/ Flossing/ Fluoride Gel/ Other

**Other observation notes**

1. What are the prevention measures included in the treatment plan?
2. Notes and Observations (patient allergy, previous hospitalisation because of asthma if dentist did not ask)

## Appendix D: Cohen’s kappa results

| **Observer** | **N of Valid Cases** | **Cohen’s kappa** | **95% CI** | **p-value** |
| --- | --- | --- | --- | --- |
| trainer * Observer 1 | 78 | 0.95 | 0.87 – 1.00 | <0.001 |
| trainer * Observer 2 | 76 | 0.95 | 0.86 – 1.00 | <0.001 |
| trainer * Observer 3 | 78 | 0.97 | 0.92 – 1.00 | <0.001 |
| trainer * Observer 4 | 70 | 0.91 | 0.81 – 1.00 | <0.001 |
| trainer * Observer 5 | 68 | 0.88 | 0.76 – 0.97 | <0.001 |

## Appendix E: Results of Intraclass Correlation Coefficient (ICC) assess the reliability between the trainer and the observers using single-rating, absolute-agreement, 2-Way mixed-effects model

| Group | cases | Complete cases | | Raters | ICC(3,1)a | | ICC(3,k)b | 95% CI |
| --- | --- | --- | --- | --- | --- | --- | --- | --- |
| Trainer + 5 observers | 10 | 8 | 6 | | | 1.00 | 1.00 | Not estimable |

## Appendix F: STROBE Statement—checklist of items that should be included in reports of observational studies

|  | **Item No** | **Recommendation** |
| --- | --- | --- |
| **Title and abstract**  (a) Indicate the study’s design with a commonly used term in the title or the abstract  (b) Provide in the abstract an informative and balanced summary of what was done and what was found | 1  Page 2 | Cross-sectional Study of Paediatric Preventive Care in Saudi Arabia |
|  |  | **Methods**: Multicentre, prospective, descriptive cross-sectional study was conducted in Jazan province, Saudi Arabia April to August 2024. Direct clinical observation allowed quantification of GDPs’ application of FV and FS in routine clinical care for children aged 14 years and younger. This was compared against current evidence-based practice guidelines.  **Results**: Thirty-five GDPs were observed delivering routine dental care to 643 children. Although 98% of children met eligibility criteria to receive FV, only 4% received it. Similarly, 74% were eligible for FS, and only 1.9% received one or more sealants. The most frequently delivered preventive measure was brushing instruction |
| **Introduction** | | |
| **Background/****rationale**  Explain the scientific background and rationale for the investigation being reported | 3  Page 4 | The shift from curative-based approaches towards a prevention focussed service is now considered one of the main objectives of the Ministry of Health for healthcare providers, with GDPs working in primary healthcare centres part of this transition. Two of the most common and effective caries preventive measures used by general dental practitioners (GDPs) are fluoride varnish (FV) and fissure sealants (FS). There is limited data on the actual practices around FV and FS use in Saudi Arabia. No studies have directly reported dentists’ use of FV. Parent-reported data indicate that only around 1.7% of children received FV applications from their GDP |
| **Objectives**  State specific objectives, including any prespecified hypotheses | 4  Page | this study aims to assess the extent to which GDPs in primary healthcare centres in Saudi Arabia provide FV and FS to eligible children 14 years old and younger, and to identify overall preventive measures they currently apply in practice. |
| **Methods** | | |
| **Study design**  Present key elements of study design early in the paper | 4  Page | multicentre, prospective, descriptive cross-sectional study |
| **Setting**  Describe the setting, locations, and relevant dates, including periods of recruitment, exposure, follow-up, and data collection | 4  Page | Study was conducted in Jazan, Saudi Arabia, between April and August 2024.  The target population were GDPs working for the Ministry of Health, in primary health care centres. |
| **Participants**  *Cross-sectional study*—Give the eligibility criteria, and the sources and methods of selection of participant | 5  Page 6 | GDPs were eligible to participate in the study if, at the time of recruitment, they were licensed to practice by the Saudi Dental Licensing Board, employed and practising in Primary Healthcare Setting within Saudi Arabia, providing full or part time clinical care, and available for the full duration of the study. GDPs who did not treat children 14 years old and younger were excluded from the study.  GDPs were identified using a database of registered Ministry of Health General Dental Practitioners working in the Jazan region. A simple random sampling approach using a random number generator selected 30 practices from the list |
|  |  |  |
| **Variables**  Clearly define all outcomes, exposures, predictors, potential confounders, and effect modifiers. Give diagnostic criteria, if applicable | 6  Page  7 | - Demographic characteristics of the GDPs and paediatric patients.  - Number of GDPs who inquired about or recorded prior FV/FS application, or who planned to apply FV/FS  - Crosstabulations to explore the proportion of eligible children who received FV and FS.  - Descriptive statistics were used to describe the distribution of GDPs offering caries prevention advice  - Practitioner- and patient-level factors (including the GDP’s years of experience and gender, and the patient’s gender and age) on the application of FV and FS for eligible children (binary outcome) |
| **Data sources/** **measurement**  For each variable of interest, give sources of data and details of methods of assessment (measurement). Describe comparability of assessment methods if there is more than one group | 6  Page  7 | The demographic characteristics of the GDPs and paediatric patients were summarised by descriptive statistics.  A frequency table was created for number of GDPs who inquired about or recorded prior FV/FS application, or who planned to apply FV/FS and crosstabulations to explore the proportion of eligible children who received FV and FS. Descriptive statistics were used to describe the distribution of GDPs offering caries prevention advice.  A generalised linear mixed model (GLMM) with a logistic link function was conducted to examine the likelihood of FV or FS being applied. The model accounted for the clustered nature of the data; multiple children treated by the same GDP. It also examined the effects of practitioner- and patient-level factors (including the GDP’s years of experience and gender, and the patient’s gender and age) on the application of FV and FS for eligible children (binary outcome) |
| **Bias**  Describe any efforts to address potential sources of bias | 13  Page | Whilst there is a possibility of behavioural modification due to the Hawthorne effect (in this case, dentists knowing there were being observed), this was minimised by having dental interns, who are routinely present in these clinics, conduct the observations. Observing practitioners for a short duration may not fully capture the scope of their preventive practices. However, the repeated observations provide valuable insight into the routine preventive care delivered to child patients. The low application rates of FV and FS for eligible children also reassure that even if the dentists were changing their behaviours to what was expected according to clinical recommendations, this was not to any great extent. |
| **Study size**  Explain how the study size was arrived at | 6  Page | The key outcomes on which the sample size calculation were based were the proportions of participating GDPs using professionally applied FV and FS respectively from previous research. This indicated that the application of FV and FS were both low at around 10%. Based on this expected prevalence and using a 95% confidence interval , a +/-10% margin of error and a population of 185 Ministry of Health general dental practitioners in Jazan, Saudi Arabia, it was calculated that observing a minimum of 30 practitioners would provide sufficient statistical power to assess FS and FV application |
| **Quantitative** **variables**  Explain how quantitative variables were handled in the analyses. If applicable, describe which groupings were chosen and why | 6-7 | Child age (years) - continuous variable (mean ± SD)  Dentist years of experience - continuous variable (mean ± SD) |
| **Statistical** **methods**  (a) Describe all statistical methods, including those used to control for confounding  (b) Describe any methods used to examine subgroups and interactions  (c) Explain how missing data were addressed  (d) (Cross-sectional study—If applicable, describe analytical methods taking account of sampling strategy  (e) Describe any sensitivity analyses | Page 6-9 | (*a*) descriptive statistics - frequency table - crosstabulations |
|  |  | (*b*) generalised linear mixed model (GLMM) with a logistic link function |
|  |  | (*c*) The missing data reported and did not included in the analysis. |
|  |  | (d) The analytical methods accounted for the sampling strategy by including GDPs as a random effect in the GLMM model to account for clustering. |
|  |  | (*e*) Likelihood ratio tests were performed to select the best-fitting model which include Akaike Information Criterion (AIC) and Bayesian Information Criterion (BIC) |
